# Pseudoaneurysm Following a Puncture of the Distal Radial Artery: A Case Report

**DOI:** 10.7759/cureus.65942

**Published:** 2024-08-01

**Authors:** Xiaofei Xie, Xiaoliang Han, Ran Li, Jinpeng Xu, Guangcheng Sun

**Affiliations:** 1 Department of Cardiology, Anhui Chest Hospital, Hefei, CHN

**Keywords:** case report, distal radial artery, pseudoaneurysm, puncture, treatment

## Abstract

Pseudoaneurysms are not uncommon in the clinic, but they have rarely been reported as a result of distal radial artery puncture. This case report is about an elderly woman who developed a pseudoaneurysm at the distal radial artery puncture site after coronary angiography via the distal radial artery. After timely treatment and long-term follow-up, the patient's hand wound gradually healed.

## Introduction

Pseudoaneurysm is a type of disease that refers to a tumor-like dilated structure formed by the destruction of the entire layer of the arterial wall due to various reasons, causing blood to overflow outside the vascular lumen and be enveloped by the surrounding tissue of the artery. After removing the catheter or sheath, a clot will form at the site of the arterial incision, usually sealing the lumen and preventing blood from continuing to flow out. If the thrombus is not sufficient, a hematoma (called a pseudoaneurysm) that communicates with the arterial lumen may form outside the artery. Pseudoaneurysms are a common complication of interventional therapy and occur due to inadequate closure of the puncture site [1]. Pseudoaneurysms usually occur after trauma, interventional therapy (a minimally invasive treatment method, through inserting a catheter into a blood vessel and delivering a guidewire or balloon to the affected area to treat the diseased vessel.), and infection. It’s a common complication of arterial catheterization [2]. The incidence of pseudoaneurysm caused by distal radial artery puncture is not high [3], but if pseudoaneurysm occurs, we should actively face it.

At present, the common approaches of cardiac interventional therapy are radial artery and distal radial artery. The probability of a pseudoaneurysm arising from a catheter passing through the radial artery is approximately 0.009% [4]. Distal radial artery puncture is less. We report a case of pseudoaneurysm in an 86-year-old woman who underwent distal radial artery puncture by cardiac angiography.

## Case presentation

**Figure 1:**
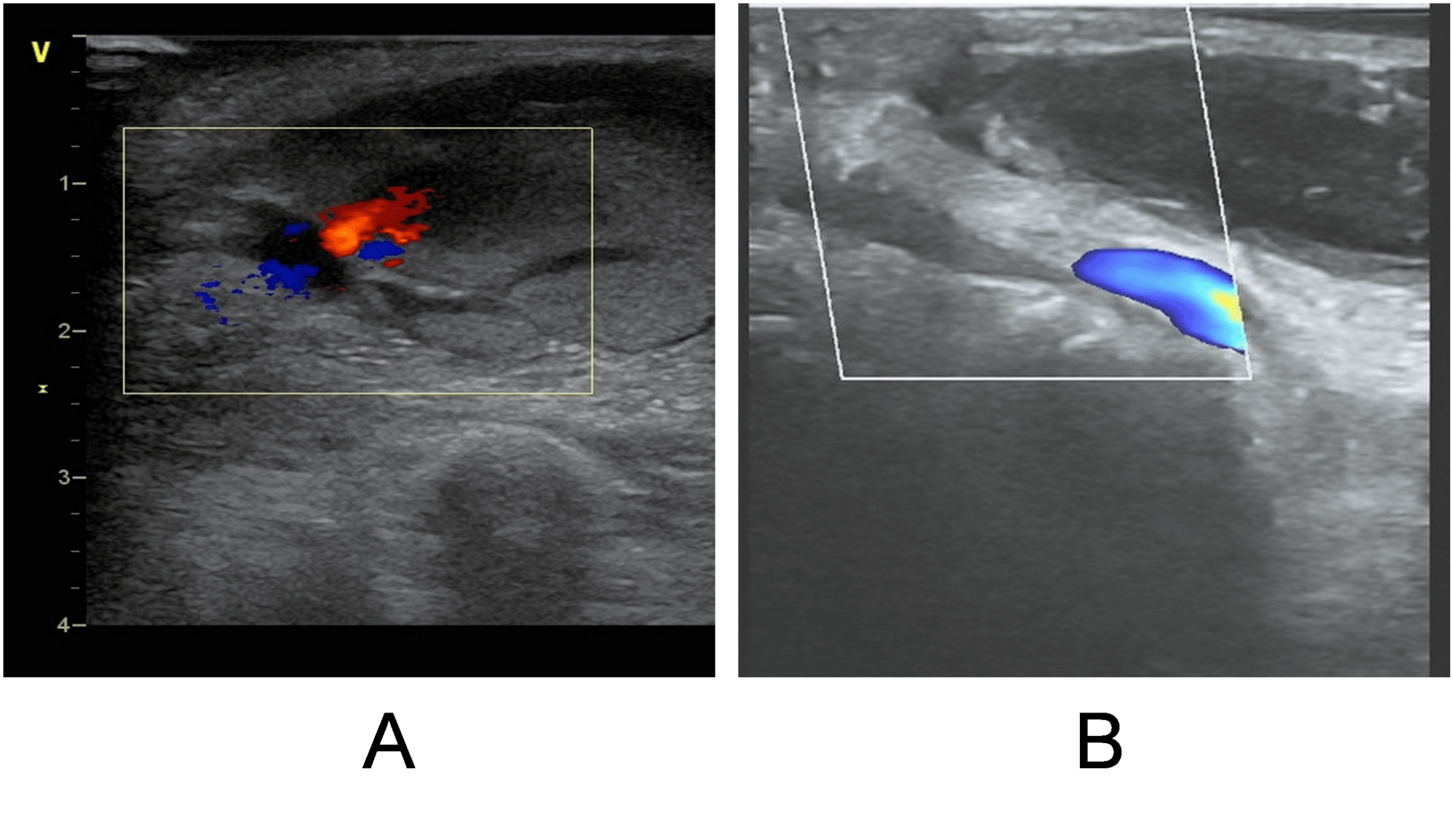
Pseudoaneurysm under ultrasound A. pseudoaneurysm before compression dressing with laceration; B. after pressure bandaging the pseudoaneurysm, the rupture disappeared.

**Figure 2:**
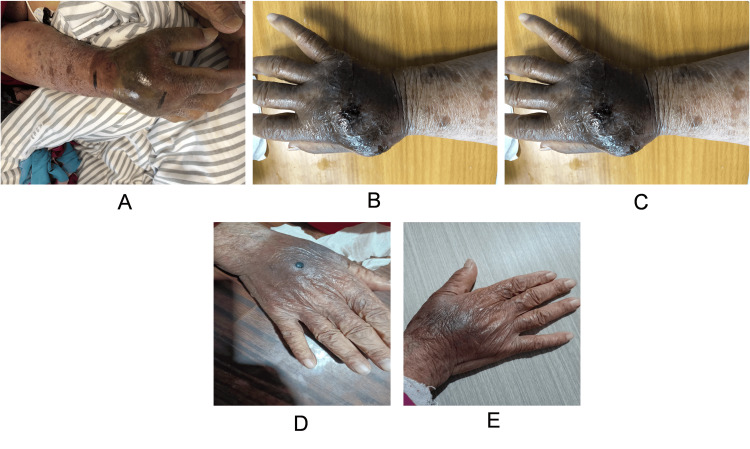
Rehabilitation of the patient's hand after treatment A. the patient's hand one day after treatment; B. the patient's hand two weeks after treatment; C. the patient's hand six weeks after treatment; D. the patient's hand nine weeks after treatment; E. the patient's hand 13 weeks after treatment

The case introduced here is of an 86-year-old Chinese woman who came to our hospital for a diagnosis of coronary heart disease. In order to understand the condition of coronary arteries, a complete coronary angiography examination will be conducted on November 30, 2023. During the operation, the right distal radial artery was selected as the puncture point for angiography. After several minutes of imaging, the sheath was removed, and pressure bandaging was performed on the right distal radial puncture site after minimally invasive intervention. After six hours of using a bandage on the patient, the pressure was relieved, but the patient's hand was swollen and painful. Ultrasound examination showed a pseudoaneurysm (Figure 1A). There was a rupture of the artery and a pseudoaneurysm (Figure 1A), and blood was flowing towards the pseudoaneurysm through the rupture. After local pressure bandaging, the rupture disappeared and the pseudoaneurysm remained (Figure 1B). After 24-hour pressure bandaging, ultrasound showed pseudoaneurysm compression, but a huge hematoma on the back of the hand was left (Figure 1B). Microorthopedic surgeons recommend washing the affected hand twice a day with alcohol, taking into account the astringent and sterilizing effects of alcohol. An erythromycin ointment was applied outside the puncture site. As the skin contracted and the hematoma was gradually absorbed, after rinsing with alcohol, vitamin E skincare cream was applied to the affected area. After a month of treatment, the patient was discharged from the hospital. After two months of follow-up, the patient's hand was healed (Figure 2).

This case report fully conforms to the Surgical CAse REport (SCARE) guideline [5]. The pseudoaneurysm is located in the right distal radial artery, and it is inoperable. Taking into account the patient's age, it was decided to observe the pseudoaneurysm by conservative treatment, regular follow-up ultrasound, and local disinfection of the skin on the back of the hand while avoiding physical and emotional stress, and after adhering to the treatment plan, the mass showed stability and decreasing size.

## Discussion

With technical innovation, the access site of coronary intervention has changed, selection of puncture site from the femoral artery to the radial artery and then to the distal radial artery [6]. Distal radial artery puncture has become a trend in cardiac interventional therapy. The arm position during the intervention is comfortable for the patients [6]. The risk of postoperative occlusion and bleeding is also significantly reduced [7]. More importantly, it can serve as a potential site for retrograde recanalization of radial artery occlusion [8]. Of course, the far radial artery puncture also has disadvantages; the puncture technique is more demanding, the puncture time is longer, and the operator radiation is larger [6].

The patient did not have any discomfort in the hand before the operation, and there was local swelling and pain after the operation. Therefore, the pseudoaneurysm was caused by improper bandaging after the operation, especially for patients with loose skin on their hands. When the distal radial artery punctures after local bandaging will cause continued bleeding and pseudoaneurysm. The presence of a pseudoaneurysm was confirmed by ultrasonography after the hand abnormality was detected. At present, common treatment methods include surgery and ultrasound-guided percutaneous thrombin injection [9,10]. During the treatment, the micro-orthopedic doctors consulted and ruled out the possibility of osteofascial compartment syndrome and suggested conservative medical treatment, local disinfection, and promotion of hemorrhage absorption. Combined with the actual situation of the patient, conservative treatment was decided. Of course, some pseudoaneurysms require surgical treatment. Another case report describes a true case of idiopathic radial artery aneurysm, where the patient underwent surgical treatment and the most severe case recovered well [11].

## Conclusions

The literature on pseudonyms is scarce. When there is local swelling and pain after a distal radial artery puncture, it may be a pseudoaneurysm. It should also be noted that different treatments are developed according to the actual situation of each patient and that measures are taken to observe cases and keep them stable; rather than performing surgery, it should also be seen as a possible treatment option in the event of such cases.
